# Periaortic Brown Adipose Tissue as a Major Determinant of [^18^F]-Fluorodeoxyglucose Vascular Uptake in Atherosclerosis-Prone, ApoE^−/−^ Mice

**DOI:** 10.1371/journal.pone.0099441

**Published:** 2014-07-23

**Authors:** Jakub Toczek, Alexis Broisat, Pascale Perret, Marie-Dominique Desruet, Daniel Fagret, Laurent M. Riou, Catherine Ghezzi

**Affiliations:** INSERM, UMR 1039, Radiopharmaceutiques Biocliniques; Université Grenoble I, La Tronche, France; University of Bologna, Italy

## Abstract

**Background:**

[^18^F]-fluorodeoxyglucose (FDG) has been suggested for the clinical and experimental imaging of inflammatory atherosclerotic lesions. Significant FDG uptake in brown adipose tissue (BAT) has been observed both in humans and mice. The objective of the present study was to investigate the influence of periaortic BAT on apolipoprotein E-deficient (apoE^−/−^) mouse atherosclerotic lesion imaging with FDG.

**Methods:**

ApoE^−/−^ mice (36±2 weeks-old) were injected with FDG (12±2 MBq). Control animals (Group A, n = 7) were injected conscious and kept awake at room temperature (24°C) throughout the accumulation period. In order to minimize tracer activity in periaortic BAT, Group B (n = 7) and C (n = 6) animals were injected under anaesthesia at 37°C and Group C animals were additionally pre-treated with propranolol. PET/CT acquisitions were performed prior to animal euthanasia and ex vivo analysis of FDG biodistribution.

**Results:**

Autoradiographic imaging indicated higher FDG uptake in atherosclerotic lesions than in the normal aortic wall (all groups, P<0.05) and the blood (all groups, P<0.01) which correlated with macrophage infiltration (R = 0.47; P<0.001). However, periaortic BAT uptake was either significantly higher (Group A, P<0.05) or similar (Group B and C, P = NS) to that observed in atherosclerotic lesions and was shown to correlate with in vivo quantified aortic FDG activity.

**Conclusion:**

Periaortic BAT FDG uptake was identified as a confounding factor while using FDG for the non-invasive imaging of mouse atherosclerotic lesions.

## Introduction

Coronary artery disease leads to myocardial infarction mostly through rupture of undetected coronary vulnerable plaques [1]. The non-invasive detection of vulnerable atherosclerotic lesions therefore represents a great yet unmet clinical challenge in the field of nuclear cardiology. Active inflammation, including monocyte/macrophage infiltration is a major criterion in the definition of vulnerable atherosclerotic lesions [2]. Plaque inflammation might be visualized with positron emission tomography (PET) using [^18^F]-fluorodeoxyglucose (FDG) through increased tracer uptake by inflammatory cells with high metabolic activity as initially shown in patients with symptomatic carotid artery disease [3]. While clinical investigation of FDG PET imaging of atherosclerosis has been an active field of research for over a decade, additional studies are still needed to establish the role of FDG–PET in atherosclerosis imaging [4], [5]. Moreover, preclinical imaging of atherosclerosis *per se* remains of great interest for preclinical and basic research. However, the potential of FDG for the non-invasive experimental assessment of atherosclerosis in the widely used apolipoprotein E-deficient (apoE^−/−^) mouse model of atherosclerosis has not been clearly defined [6]–[11].

The brown adipose tissue (BAT) is involved in nonshivering thermogenesis under sympathetic nervous system control and display varying and potentially high levels of metabolic activity depending upon the energetic metabolic status, anaesthetic conditions, pharmacological interventions and external temperature [12]. Accordingly, FDG uptake in brown adipose tissue has been reported to reach significant levels in humans as well as in mice [7], [13], [14]. A periaortic adipose tissue with virtually identical features to those of brown adipose tissue has been identified and studied [15]. Periaortic BAT is located in the immediate vicinity of aortic atherosclerotic lesions and has recently been assigned an atheroprotective role through adaptive thermogenesis [16]. Despite its anatomical proximity with atherosclerotic lesions, the potential impact of periaortic BAT on noninvasive FDG vascular imaging of atherosclerotic plaques has not been evaluated yet. Therefore, the objective of the present study was to investigate the potential confounding effect of periaortic BAT on apoE^−/−^ mouse aortic atherosclerotic lesion imaging with FDG under experimental conditions affecting BAT metabolic activity. FDG aortic uptake in apoE^−/−^ mice was studied *in vivo* using a state-of-the-art, small-animal dedicated PET/CT camera and *ex vivo* using autoradiographic imaging of the aortic arch in order to discriminate atherosclerotic lesion from periaortic BAT tracer uptake.

## Materials and Methods

### Ethical statement

All animal procedures were conducted under the approval of the animal experimentation ethics committee ComEth Grenoble (authorization # 151_LER-U1039-LR-01). All efforts were made to minimize suffering.

### Animal study

**Figure 1:**
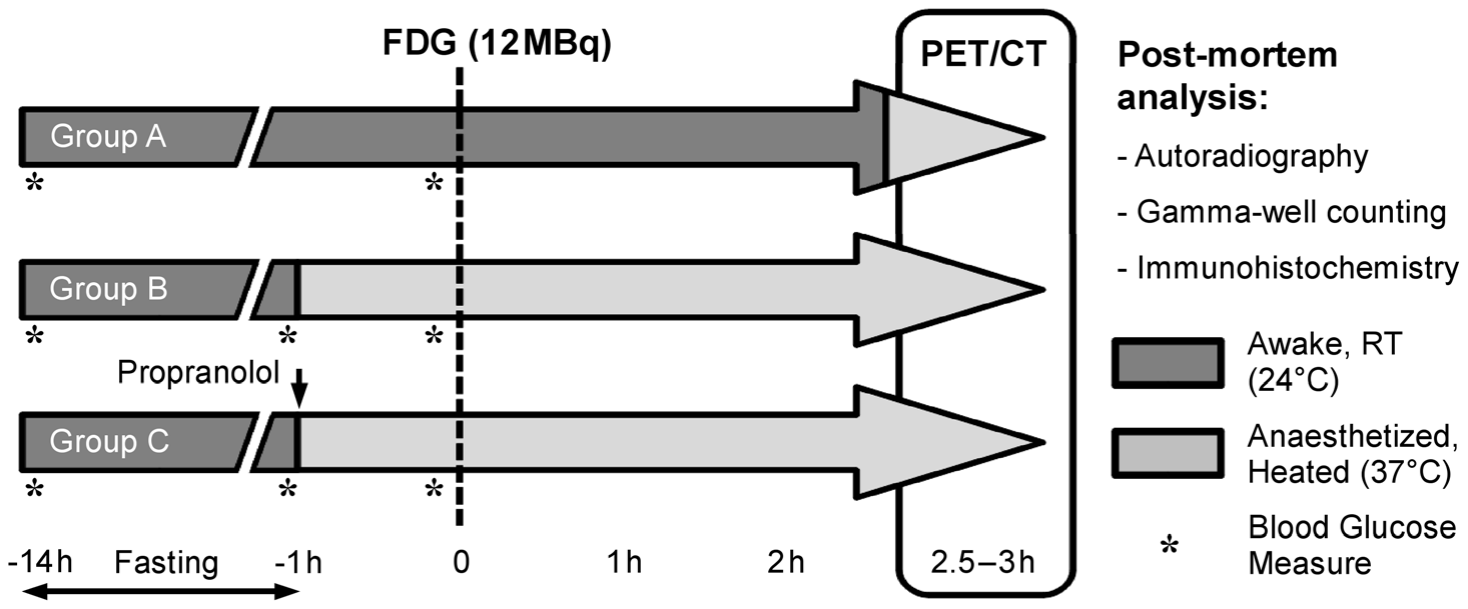
Experimental protocol. RT, room temperature.

The experimental procedure is summarized in Figure 1. Twenty (20) female apoE^−/−^ mice aged 36±2 weeks and fed a high-fat western type chow containing 0.05% cholesterol (Safe, Augy, France) for 24±6 weeks were used. The mice were housed under controlled humidity, temperature, and light cycle conditions. All animals were kept at 24°C and fasted for 14 h before the imaging protocol. After 13 h of fasting and 1 h before FDG injection, the animals were randomly assigned to 1 of 3 experimental groups, namely Group A, B and C. Animals from Group A (n = 7) were not anesthetized while Group B (n = 7) and Group C (n = 6) animals were anesthetized using intraperitoneal (i.p.) sodium pentobarbital (0.055 mg·kg^−1^) and kept under anesthesia with an i.p. infusion of 0.020 mg·kg^−1^·h^−1^ sodium pentobarbital for 4 h while kept in a 37°C warmed bed with oxygen supplementation. In addition, Group C animals were injected with DL-propranolol (Sigma-Aldrich) 10 mg·kg^−1^ i.p. following anesthesia induction. FDG (12±2 MBq) was injected in the tail vein 1 h after the induction of anesthesia in Group B and C animals whereas Group A animals were injected conscious at the same time point. Animal weight and blood glucose (Accu-check performa, Roche Diagnostics) were assessed before the fasting period as well as before FDG injection in all 3 experimental groups while an additional assessment of glycemia was performed immediately prior to anesthesia induction in Groups B and C. Group A animals were anesthetized for the purpose of image acquisition at 2.25 h following FDG injection (i.e. 0.25 h prior to PET image acquisition, see below) using isoflurane 2% in an oxygen-supplemented gas mixture. FDG image acquisition was started 2.5 h following tracer injection and lasted for 0.5 h in all animals from groups A, B, and C (see details below). All animals were euthanized 3 h following FDG injection through cervical dislocation while still under deep anesthesia.

### PET/CT imaging

Dual modality PET/CT acquisitions were performed using a small animal dedicated system (nanoPET/CT, Bioscan) and proprietary software (Nucline 1.07, Bioscan). A thirty (30) min-long PET acquisition was performed from 2.5 to 3 h p.i. with 1-3 coincidence mode and image reconstruction was performed using constructor's OSEM algorithm. A CT acquisition was then performed using 180 projections (600 ms each) at 45 kVp and reconstructed using dedicated software (Nucline 1.07, Bioscan). PET images were quantified using InVivoScope 2.0 software (Bioscan) and 2 volumes of interest (VOIs) were drawn. A first spherical volume of interest (radius: 0.5 mm) was centered on aortic arch lesions as identified by CT signal enhancement [17] and a second VOI matched the prevertebral course of the thoracic aorta above the diaphragm muscle. Activity concentration in the VOI was expressed as mean standardized uptake value (SUV_mean_), i.e. tissue activity concentration (MBq·mL^−1^)/injected dose (MBq) · body weight (g).

### *Ex vivo* protocol

After euthanasia, multiple tissues samples were harvested, weighed and their activity was measured (Cobra II Auto-Gamma counter, Packard). Tissue activities from gamma-well counting were corrected for decay and tissue activity concentrations were then expressed as a differential uptake ratios (DUR) corresponding to tissue activity concentration (MBq·g^−1^)/injected dose (MBq) · body weight (g). The proximal thoracic aorta was harvested and deep-frozen in −40°C cooled 2-methylbutane along with other tissues samples of known activity concentration. Twenty (20) µm-thick sections were obtained (HM505E, Microm), exposed on an autoradiographic film (08SR2025, Fujifilm) and read using a phosphorimager (BAS-5000, Fujifilm). Exposed tissues sections were stained with HES trichrome coloration (Haematoxylin, Erythrosine, Saffron) while adjacent 8 µm-thick tissue sections were used for immunohistochemistry.

### Immunohistochemistry

Macrophage and BAT immunostainings were performed on 8 µm-thick frozen sections using anti-F4/80 and anti-UCP-1 antibodies, respectively. Briefly, endogenous peroxidases were blocked with hydrogen peroxide on −20°C acetone fixed slices. Following a blocking step at room temperature, the primary antibody was applied on the tissue sections overnight at 4°C (anti-F4/80, 1∶200, 14-4801-85 (BM8), eBioscience; and anti-UCP-1, 1∶500, sc-6528 (C17), Santa Cruz Biotechnology). The appropriate biotinylated secondary antibody (Jackson ImmunoResearch) was incubated (1 h at RT) prior to revelation with serial incubation of ABC and DAB (Vector Labs) for UCP-1 staining and Simple Stain Mouse MAX PO (rat) kit (N-Histofine) for macrophage staining and counterstaining with hematoxylin was then performed.

### *Ex vivo* image analysis

Regions of interest (ROIs) corresponding to the atherosclerotic lesion, the periaortic BAT and the lesion-free aortic wall were drawn on the digitized HES-stained sections (Discovery.V8, Zeiss). The histological relevance of the ROIs was assessed by means of comparison with an adjacent section immunostained for macrophages and BAT. Following semi-automated landmark-based coregistration, ROIs were used to quantify tracer activity on the autoradiographic image (Gimp 2.6.8 and ImageJ 1.46). The same process was used to quantify activity of reference tissues samples with known amounts of radioactivity, which allowed the conversion of intensity values into MBq.g^−1^. Tissue sections stained for macrophages were digitized (Discovery.V8, Zeiss) and used to assess macrophage infiltration in individual lesions through the evaluation of the F4/80 positive-to-total lesion area ratio (Gimp 2.6.8 and ImageJ 1.46).

### Statistical analysis

Numeric parameters were expressed as mean ± SD. Data were tested for homogeneity of variance using Fisher's F-test. Mann and Whitney's U test or Student's t-test were performed to compare data sets. Pearson's product-moment correlation was calculated to assess the significance of linear regressions (R 3.0/R Commander 2.0 and LibreOffice 4.0). A *P* value of less than 0.05 was considered statistically significant.

## Results

### Blood glucose and body weight

The results are presented in Table 1. Overnight fasting resulted in a significant decrease in blood glucose in all experimental groups (% decrease, 45±12, 45±12, and 41±16 for Group A, B, and C, respectively, *P* =  NS between Groups) while body weight decreased by 7±1%, 7±2%, and 9±2% in Group A, B, and C, respectively (*P* =  NS between Groups). In addition, 1 h of anesthesia did not modify significantly blood glucose in group B animals (4.9±0.4 vs. 4.5±0.9 mmol·L^−1^, *P* =  NS), whereas propranolol injection in anesthetized animals from Group C resulted in a significant increase from 4.8±0.3 mmol·L^−1^ to 6.3±0.8 mmol·L^−1^ (*P*<0.01).

**Table 1:** Blood glucose levels.

	Fed	Fasting	
		Anesthesia	FDG injection
**Group A**	8.5±0.6	N/A	4.6±0.9†
**Group B**	8.3±1.4	4.5±0.9†	4.9±0.4* †
**Group C**	8.5±0.6	4.8±0.3†	6.3±0.8† ‡

Data are mmol·L^−1^.

*, *P*<0.05 vs. Group C;

†, *P*<0.05 vs. Fed;

‡, *P*<0.05 vs. Fasting/Anesthesia.

### *Ex vivo* determination of FDG biodistribution

The *ex vivo*-determined FDG biodistibution in the thoracic aorta and surrounding tissues is presented in Table 2. Blood activity was significantly lower in Group A as compared with Group B (*P*<0.01), but not with Group C (*P* = 0.15), with Group B and Group C blood activities being comparable (*P* = 0.22). Heart uptake on one hand and atherosclerotic lesion uptake on the other hand were comparable in Group A and B animals, (*P* =  NS for both) while Group C animals displayed increased cardiac FDG uptake (DUR: 4.61±2.62, *P*<0.05 vs. lesion and Group A and *P* = 0.07 vs. Group B). Diaphragm muscle uptake was higher or tended to be higher in Group A than in Groups B and C (*P* = 0.07 vs. Group B and *P*<0.05 vs. Group C). Interscapular BAT FDG uptake was higher in group A compared to groups B and C (*P*<0.05 for both). A positive correlation was observed in FDG uptake between interscapular and periaortic BAT (*R* = 0.90, *P*<0.001).

**Table 2:** *Ex vivo* assessment of FDG biodistribution.

	Group A	Group B	Group C
**Autoradiography**			
Atheroscleroctic lesion	1.57±0.79§	1.50±0.74	1.39±0.28
Lesion-free aortic wall	0.54±0.24‡ §	0.53±0.25‡ §	0.62±0.26‡
Periaortic BAT	4.88±2.85‡	1.57±1.05*	1.44±0.99*
**Tissues samples**			
Blood	0.18±0.04‡ §	0.34±0.132* ‡ §	0.22±0.07‡ §
Heart	1.89 ±0.62§	1.96±1.33	4.64±2.87† ‡ §
Thymus	1.08±0.27§	1.32±0.68	1.88±1.20
Lymph nodes	0.84±0.34§	1.75±0.94*	1.36±0.31*
Lungs	0.85±0.20‡ §	1.47±0.40*	1.28±0.36*
Liver	0.29±0.06‡ §	0.59±0.13* ‡ §	0.50±0.07* ‡ §
Diaphragm muscle	2.97±1.62	0.94±1.03*	0.79±0.30* ‡
Interscapular BAT	10.32±7.04‡	1.74±0.97*	1.69±0.81*

Data are DUR.

*, *P*<0.05 vs. Group A;

†, *P*<0.05 vs. Group B;

‡, *P*<0.05 vs. Atheroscleroctic lesion;

§, *P*<0.05 vs. Periaortic BAT.

#### FDG uptake in the thoracic aorta

**Figure 2:**
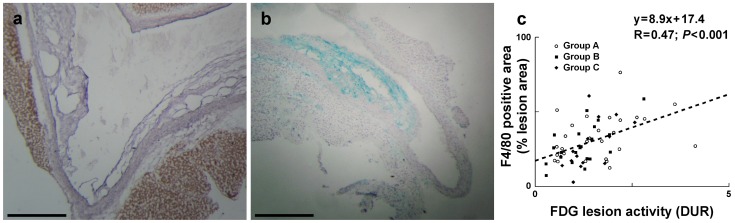
Immunohistochemical analysis of the aortic arch. Immunohistochemical stainings of UCP-1 (a) and F4/80 (b) were performed on tissue sections from the aortic arch of apoE^−/−^ mice. UCP-1 staining was used to control the identity of the periaortic adipose tissue. F4/80 macrophage staining was used to quantify atheromatous lesions macrophage infiltration. Scale bar: 200 µm. A positive correlation was observed between macrophage infiltration and FDG uptake assessed through autoradiographic images quantification performed on adjacent tissue sections (c).

**Figure 3:**
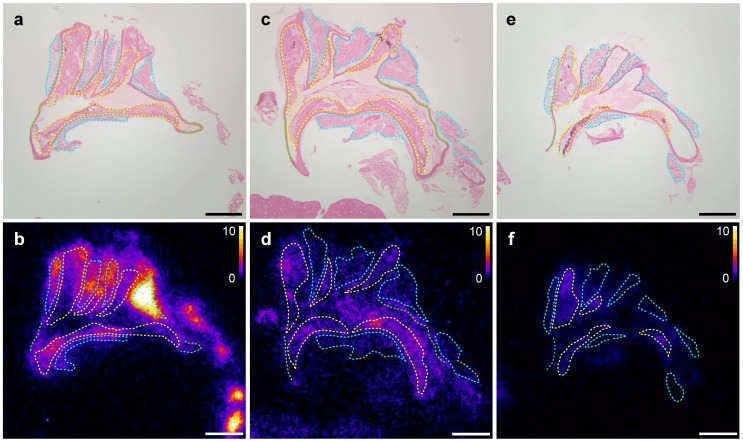
Autoradiographic analysis of FDG uptake in the aortic arch. Autoradiographic analysis of FDG uptake in aortic arch from apoE^−/−^ mice from Group A (a, b), B (c, d) and C (e, f). The exposed section were stained with HES trichrome staining (a, c, e), regions of interest were defined (yellow: lesion; green: lesion-free aortic wall; teal: perioartic BAT) and used to quantify the autoradiographic acquisition (b, d, f). Scale bar: 500 µm; autoradiographic images intensity is expressed as DUR.

**Figure 4:**
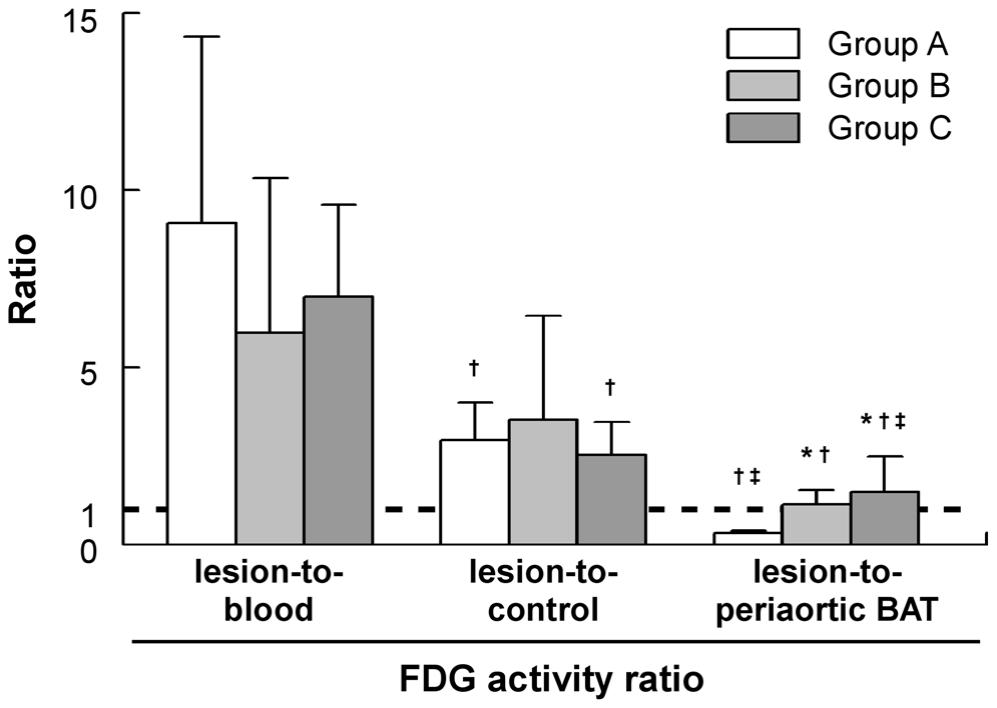
*Ex vivo*-determined FDG activity ratios. FDG lesion-to-blood, lesion-to-control and lesion-to periaortic BAT activity ratios from *ex vivo* imaging and biodistribution experiments. *, *P*<0.05 vs. Group A; †, *P*<0.05 vs. lesion-to-blood; ‡, *P*<0.05 vs. lesion-to-control.

The adipose tissue surrounding the superior and posterior mediastinum large arteries was identified as periaortic brown adipose tissue (BAT) as indicated by positive anti-UCP-1 immunohistochemical staining (Figure 2A). Macrophage infiltration in aortic atherosclerotic lesions was also demonstrated by F4/80 immunostaining (Figure 2B) and represented approximately 30% of lesion area with no statistical difference between experimental groups. Shown in Figure 3 are representative histological stainings of longitudinal sections of the aortic arch and surrounding tissues together with the corresponding autoradiographic images of FDG activity from Group A, B, and C animals and allowing the distinction between atherosclerotic lesion, normal wall, and periaortic BAT FDG uptake. Quantification of autoradiographic images is provided in Table 2 (see above). The results first indicated that macrophage infiltration in atherosclerotic lesions correlated positively with lesional FDG uptake (*R* = 0.47; *P*<0.001) (Figure 2C). In addition, overall FDG atherosclerotic lesion uptake was not statistically different between experimental groups. Tracer activity in the lesion-free, normal aortic wall was also comparable between experimental groups (*P* =  NS), whereas periaortic BAT tracer activity was significantly lower in Groups B and C in comparison with Group A. FDG uptake in atherosclerotic lesions was not statistically different from that observed in the periaortic BAT in Groups B and C (*P* = 0.90 and *P* = 0.91, respectively) whereas accumulation at room temperature in Group A animals resulted in higher uptake in periaortic BAT than in the atherosclerotic lesion (*P*<0.05). Lesion-to-blood and lesion-to-control aorta ratios were comparable between Groups A, B and C (*P* =  NS). Lesion-to-periaortic BAT uptake ratios were of 0.3±0.1, 1.1±0.4 and 1.5±1.0 for Groups A, B, and C respectively, with the higher periaortic BAT FDG activity in Group A resulting in a significantly lower lesion-to-periaortic BAT ratio than in Group B and Group C (*P*<0.05) (Figure 4).

### PET/CT imaging

**Figure 5:**
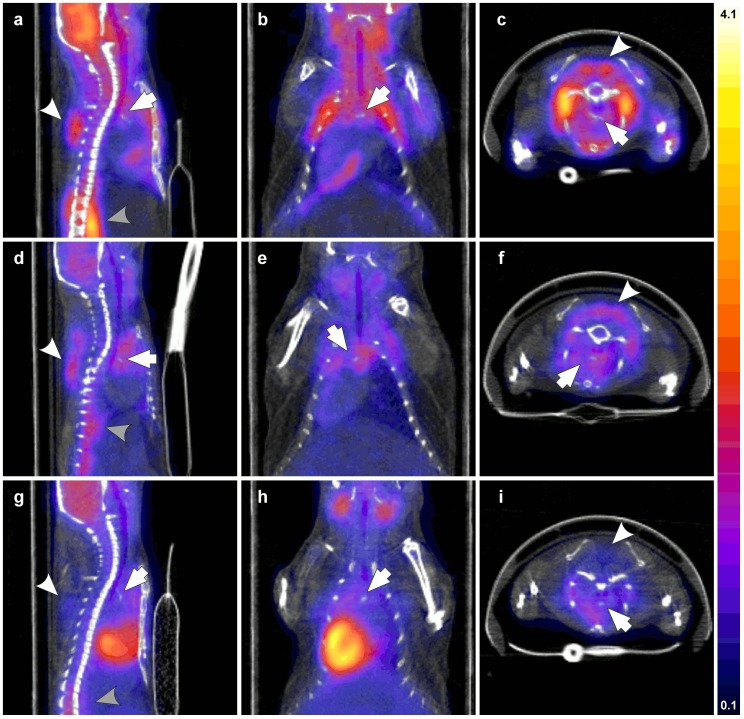
PET/CT Imaging. Fusion PET/CT images acquired 2.5 to 3 h after the injection of FDG in apoE^−/−^ mice from Group A (a, b, c), B (d, e, f) and C (g, h, i) groups in sagittal (a, d, g), coronal (b, e, h) and transverse (c, f, i) views centered on the aortic arch (arrows). The interscapular BAT (white arrowheads) and the diaphragm muscle (gray arrowheads) were identified on those views. Scale set at 0.1 to 4.1 SUV for all images.

Representative PET/CT images of FDG uptake in apoE^−/−^ mice are presented in Figure 5, with sagittal, coronal, and transverse views encompassing the aortic arch area as identified using anatomical landmarks from the CT image. Results from image quantification are presented in Table 3. FDG uptake by interscapular BAT was readily observable on images (Figure 5, white arrowheads), with significantly higher mean tracer activity in Group A than in Group B and Group C. Tracer uptake was also noticed at the site of diaphragm aortic insertion (grey arrowheads), with significantly higher activity in Group A than in Groups B and C. FDG activity was observed in the aortic arch (arrows) and thoracic aorta in all experimental groups. Image quantification indicated that significantly higher activity was observed in the aortic arch and thoracic aorta of Group A than in those of Group B animals. Although not significant, a strong trend towards lower activity in Group C, anesthetized and propranolol-treated animals maintained at 37°C was also observed in comparison with Group A (*P* = 0.09 and 0.07 for the aortic arch and thoracic aorta, respectively). Finally, myocardial FDG uptake was significantly higher in Group C animals than in Groups A and B.

**Table 3:** In vivo image quantification.

	Group A	Group B	Group C
Aortic arch	1.4±0.5	0.9±0.2*	1.0±0.2 (*P* = 0.09)
Thoracic aorta	1.2±0.3	0.8±0.1†	0.9±0.1 (*P* = 0.07)
Interscapular BAT	5.2±2.4	1.1±0.8†	1.1±0.4†
Diaphragm muscle	2.7±0.7	1.0±0.3†	0.9±0.2†

Data are SUV_mean_.

*, *P*<0.05 vs. Group A;

†, *P*<0.01 vs. Group A. Given in parentheses are *P* values for the comparison of Group C vs. Group A.

**Figure 6:**
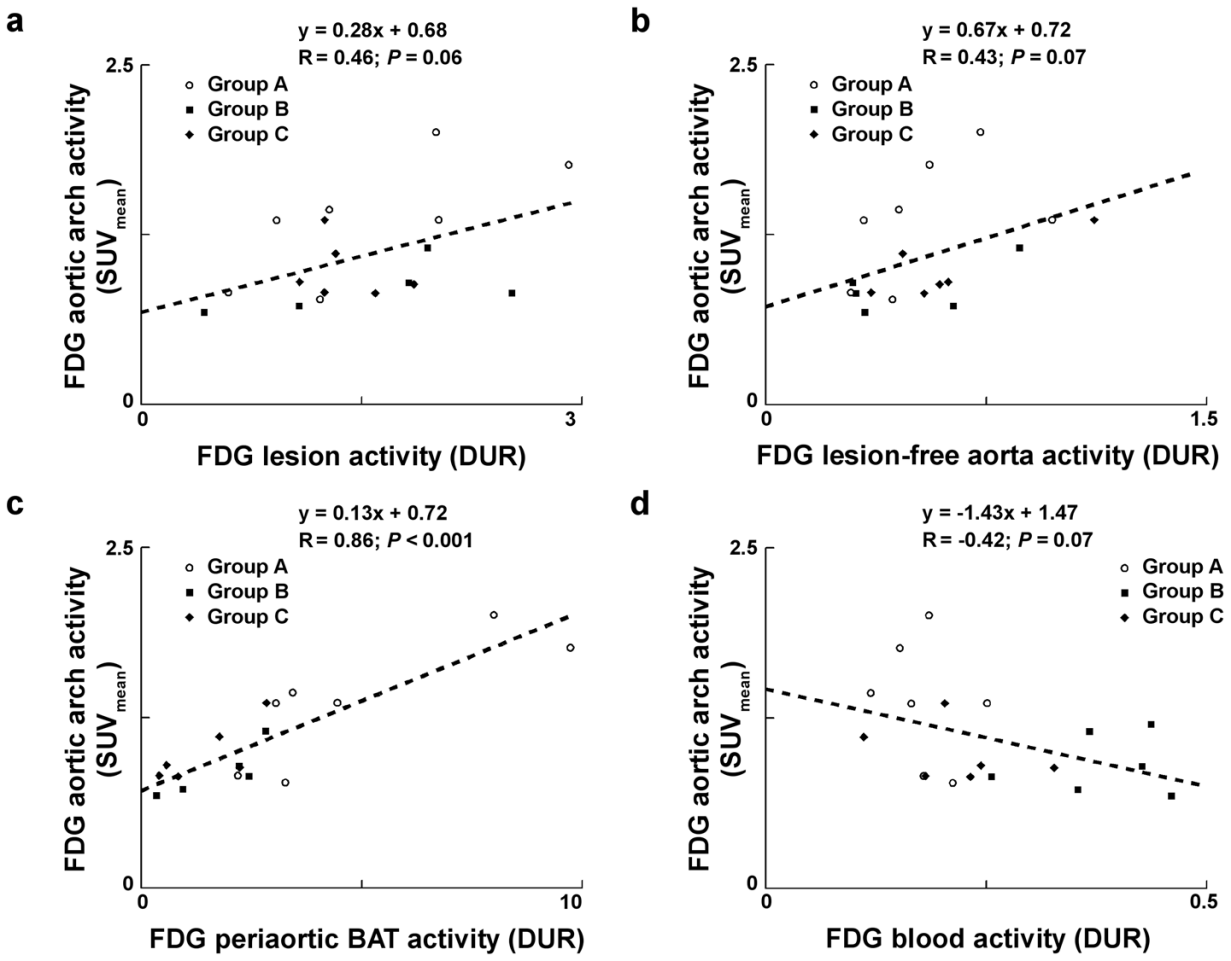
Correlation between *in vivo* PET/CT imaging and *ex vivo* biodistribution data. Correlations between FDG activity from *in vivo* PET image quantification using a VOI centered on the aortic arch and *ex vivo*-determined FDG atherosclerotic lesion activity (a), FDG lesion-free aortic activity (b), FDG periaortic BAT activity (c) and FDG blood activity (d).

Correlation between *in vivo* PET/CT imaging and *ex vivo* biodistribution data are presented in Figure 6. Periaortic BAT uptake positively correlated to SUV_mean_ from *in vivo* PET images at the level of the aortic arch (*R* = 0.86, *P*<0.001) while atherosclerotic lesion, normal wall, and blood tracer activities were not significantly correlated with values from *in vivo* image quantification (*P* =  NS).

## Discussion

The main result from the present study was that quantification of *in vivo* high resolution, small animal-dedicated PET/CT images of aortic FDG uptake in the apoE^−/−^ murine model of atherosclerosis was dependent upon periaortic brown adipose tissue FDG uptake despite favorable, >>1 lesion-to-blood and lesion-to-lesion-free aortic wall tracer activity ratios as assessed by *ex vivo* analysis. In our experimental conditions, including the use of temperatures exceeding thermal neutrality and propranolol, the significant FDG uptake in periaortic BAT represents a confounding factor for the non invasive imaging of FDG uptake in murine aortic atherosclerotic lesions.

### Metabolic conditions and FDG biodistribution

The metabolic status can greatly influence FDG biodistribution. Animal handling was therefore optimized in order to maximize FDG atherosclerotic lesion uptake in contrast with surrounding tissues. All animals were fasted overnight in order to minimize blood glucose level before the experimentation. Anesthesia induction prior to the injection of FDG and maintenance throughout the accumulation period allowed the lowering of FDG uptake in tissues minimally stimulated in unconscious animals such as the brain and muscles. Sodium pentobarbital anesthesia was chosen over ketamine/xylazine since marked hyperglycemia has been observed with the latter [18]. Isoflurane was also discarded during the FDG accumulation period since it has been shown to increase FDG myocardial uptake [19]. Overall, sodium pentobarbital was expected to have minimal effects on FDG kinetics [20].

As expected from BAT physiology [12], heating the animal over its thermoneutral zone reduced FDG uptake in both the interscapular and periaortic BAT. BAT metabolism is mainly driven through β3-adrenergic activation [21]. An attempt to further reduce BAT FDG uptake was therefore performed using the non-selective β-blocker propranolol using previously described experimental conditions [22]. However, BAT FDG uptake was not further reduced when compared to animals not injected with propranolol, probably due to the fact that warming by itself almost completely inhibited BAT metabolism. In addition, an increase in blood glucose level in the presence of propranolol was observed at the time of tracer injection, which was likely due to the worsening of glycemic control induced by the β-blocker [23] in association with the transient hyperglycemia induced by sodium pentobarbital anesthesia [20]. The anesthesia and propranolol-induced increase in blood glucose level might in turn have caused the observed increase in myocardial FDG uptake reflective of increased myocardial glucose metabolism in animals injected with propranolol.

### Autoradiographic imaging for the distinction between lesion, normal wall, and periaortic BAT FDG uptake

#### Atherosclerotic lesion uptake

FDG lesion-to-lesion-free aortic wall uptake ratio was similar to previously reported values from autoradiographic studies performed in murine models, either on tissues sections [24] or on aorta en face [6]. FDG uptake in lesions positively correlated with macrophage infiltration. Using a similar methodology, such a correlation was already reported in apoE^−/−^ mice when the evaluation was performed at 2 h p.i. [25], while no correlation was found at an earlier time point of 20 min p.i. [6]. Such a correlation is in accordance with the hypothesis of macrophages representing the main source of FDG uptake within plaques, while not excluding that others parameters such as hypoxia [26] or macrophages differentiation [27] might significantly influence FDG uptake within lesions.

#### Periaortic BAT uptake

Similarly to the above mentioned results on interscapular BAT FDG activity from biodistribution and *in vivo* imaging studies, autoradiographic imaging readily showed that periaortic BAT FDG uptake could be lowered using appropriate animal handling. Anesthesia and animal heating allowed a significant reduction of FDG uptake in periaortic BAT which was not further decreased with the use of propranolol. However, qualitative and quantitative autoradiographic image analysis indicated that the lowest achievable periaortic BAT tracer activity remained at a similar level to that measured in adjacent atherosclerotic lesions.

### *In vivo* imaging

Rudd *et al*. have suggested that a potential reason accounting for the initial failure to identify atherosclerotic lesions by FDG PET imaging of apoE^−/−^ mouse might be that the 1 h time point chosen for image acquisition did not allow tracer accumulation into atherosclerotic lesions [8]. Tracer accumulation was therefore allowed for 3 h in the present study, in accordance with the experimental conditions described in recent studies [10]. The effect of animal handling on BAT metabolism was confirmed by *in vivo* imaging indicating varying interscapular BAT FDG uptake, with significantly lower interscapular BAT activity in anesthetized, thermoregulated animals.

The use of a state-of-the-art, small-animal dedicated PET/CT camera prevents the accumulation of FDG in the interscapular region from representing a confounding factor for atherosclerotic lesion imaging as previously suggested [7]. On the other hand, given the moderate size of mouse atherosclerotic lesions in comparison with the spatial resolution of state-of-the-art small-animal dedicated PET systems, partial volume effect is necessarily involved while performing mouse atherosclerotic lesion imaging. This implies that while evaluating FDG uptake of lesions from the aortic sinus, the elevated myocardial FDG uptake represents a major confounding factor; the lesions being present at this anatomical location are therefore not used in studies specifically using FDG [28]. Similarly, the observed activity quantified from a VOI drawn on *in vivo* PET images and centered on atherosclerotic lesions of the aortic arch as identified by signal enhancement on non-contrast CT acquisitions [17] therefore represents FDG uptake in atherosclerotic lesions as well as in tissues immediately adjacent to the atherosclerotic lesions, i.e. the blood, lesion-free aortic wall and periaortic BAT. The results of the present study indicated that SUV_mean_ values measured in the aortic arch on *in vivo* PET images positively correlated with the *ex vivo* determined FDG uptake in periaortic BAT using high resolution *ex vivo* autoradiographic imaging. More precisely, the modulation of periaortic BAT FDG uptake in thermoregulated Group B animals in comparison with control Group A animals as assessed by *ex vivo* analysis resulted in a significantly lower *in vivo* FDG uptake in the VOI encompassing the aortic atherosclerotic lesions. Although lower FDG aortic activity was also observed in Group C than in Group A, the difference did not reach statistical significance most likely due to the fact that propranolol treatment in Group C animals induced a transient increase in glycemia that might have favored FDG uptake by glucose utilizing tissues (see Metabolic condition and FDG biodistribution above).

### Experimental and clinical implications

The present study indicated that the findings from *ex vivo* autoradiographic image analysis indicating higher or similar FDG activity in periaortic BAT than in atherosclerotic lesions translated into significant differences in tracer activity upon *in vivo* image quantification. Periaortic BAT therefore represents a confounding factor for the *in vivo* assessment of FDG atherosclerotic lesion activity in the aorta of apoE^−/−^ mice. Such a limitation to the use of FDG for aortic atherosclerosis imaging should be expected in all mouse models of atherosclerosis. However, recently published data suggested that perivascular adipose tissue around the murine common carotid artery did not significantly participate to overall vascular tracer uptake [11]. Mouse carotid lesions might therefore be more suitable than aortic lesions for mouse atherosclerosis imaging with FDG even though initial experiments of total carotid ligation in apoE^−/−^ mice did not allow to identify FDG uptake in the corresponding lesions [7].

Clinically, BAT activation was initially identified using FDG PET/CT imaging in the supraclavicular area of human adults [29]. BAT activation depends on multiple factors including subject age, adiposity and outdoor temperature [14], [30]. Of note, mediastinal BAT activation was also observed in human adults, including in the periaortic and pericardial regions, the latter being in the continuity of pericoronary adipose tissue [31]. Adipose tissue in the vicinity of carotid arteries was also identified as a potential source of FDG uptake [32]. In addition to mouse, perivascular BAT activation could therefore represent a potential confounding factor for atherosclerosis imaging using FDG in humans. However, the retrospective incidence of BAT detection with FDG was shown to be modest since it ranged from 1.7 to 9.3% [30]. In addition, human perivascular coronary adipocytes are white rather than brown adipocytes [33]. Overall, FDG uptake by human adipose tissue should therefore represent a lower bias than that demonstrated in the present study in a preclinical mouse model of atherosclerosis focused on aortic lesions imaging. FDG PET/CT imaging of carotid arteries in the clinical settings is a relatively well-established methodology with good short-term reproducibility [34] which is now used in clinical trials as an end-point to evaluate the effect of therapeutic interventions [35], [36]. On the other hand, the elevated FDG myocardial uptake represents a major pitfall for coronary atherosclerosis imaging, despite the use of fasting, low-carbohydrate/high fat meals, or heparin to minimize myocardial activity [37], [38].

## Conclusion

In our experimental conditions, FDG uptake in the periaortic BAT of apoE^−/−^ mice was either higher or similar to that observed in the immediately adjacent atherosclerotic lesion depending upon the metabolic status of the animals. These differences in periaortic BAT FDG activity as assessed from *ex vivo* high resolution autoradiographic images translated into corresponding variations in tracer activity as assessed from *in vivo* PET image quantification. Periaortic BAT uptake therefore represents a confounding factor while using FDG imaging for the non-invasive assessment of mouse atherosclerotic lesions, thereby emphasizing the need to carefully design preclinical studies using FDG for the evaluation of lesion inflammation in this animal model.

## Supporting Information

Data S1 Study Data.* All study data, including animals age, weight, blood glucose level, FDG injected dose and FDG biodistribution determined *ex vivo *in tissue samples and using autoradiographic analysis and *in vivo *using PET image quantification, are presented in the supporting file.
(XLSX)
